# Serum S100B and Clinical Outcomes After Spontaneous Subarachnoid Hemorrhage: A Prospective Observational Study

**DOI:** 10.1007/s12035-026-06202-2

**Published:** 2026-09-21

**Authors:** Anna Maria Auricchio, Silvia Baroni, Michele Nichelatti, Giulia Napoli, Francesco Calvanese, Giovanni Maria Ceccarelli, Renata Martinelli, Grazia Menna, Marco Obersnel, Luca Scarcia, Andrea Alexandre, Anselmo Caricato, Carmelo Lucio Sturiale, Alessio Albanese, Enrico Marchese, Francesco Doglietto, Andrea Urbani, Alessandro Olivi, Giuseppe Maria Della Pepa

**Affiliations:** 1 Neurosurgery, University Hospital Foundation A. Gemelli IRCCS, Catholic University of the Sacred Heart, Largo Agostino Gemelli, 8, 00168 Rome, Italy; 2 Neurosurgery, University Hospital of Pisa, AOUP, Via Paradisa 2, 56124 Pisa, Italy; 3 Laboratory and Haematological Sciences, University Hospital Foundation A. Gemelli IRCCS, Catholic University of the Sacred Heart, Rome, Italy; 4 Basic Biotechnological Sciences, Intensive Care and Perioperative Clinics Research, University Hospital Foundation A. Gemelli IRCCS, Catholic University of the Sacred Heart, Rome, Italy; 5 Complex Structure of Clinical Research and Innovation, ASST Great Metropolitan Hospital Niguarda, Milan, Italy; 6 Neurosurgery Department, ASST Papa Giovanni XXIII Hospital, Bergamo, Italy; 7 Neuroradiology Unit, Henri Mondor Hospital, Creteil, France; 8 Diagnostic Radiology, University Hospital Foundation A. Gemelli IRCCS, Catholic University of the Sacred Heart, Rome, Italy; 9 Emergency, Anesthesiology and Intensive Care Medicine, University Hospital Foundation A. Gemelli IRCCS, Catholic University of the Sacred Heart, Rome, Italy

**Keywords:** Subarachnoid hemorrhage, DCI, Biomarkers, Complications

## Abstract

This study aims to investigate the prognostic value of serum S100B protein in patients with spontaneous subarachnoid hemorrhage (sSAH), specifically in predicting mortality and functional outcomes. A prospective observational study was conducted on 108 consecutive sSAH patients admitted between January 2022 and August 2024. Serum S100B levels were measured at 24 h (T0), 72 h (T1), and 7 days (T2) post-admission. Clinical outcomes, including the modified Rankin Scale (mRS) at 14 days and 3 months, as well as complications such as delayed cerebral ischemia (DCI) and systemic adverse events, were recorded. Associations were analyzed using logistic regression and explored using random forest with repeated cross-validation. Higher early S100B levels were independently associated with worse functional outcome and higher mortality. S100B at T1 was associated with delayed cerebral ischemia (DCI) in univariate analysis, but this association did not persist after multivariable adjustment for clinical severity. Random forest analyses supported the prognostic value of early S100B measurements for functional outcome; S100B at T0 and T1 emerged as the most influential predictors. No significant association was found between S100B levels and systemic complications. S100B is a promising early biomarker for predicting mortality and functional outcomes in sSAH, whereas its independent association with DCI and its predictive value for systemic complications appear limited. Larger validation studies are warranted before biomarker-guided clinical protocols can be considered.

**Supplementary Information:**

The online version contains supplementary material available at https://doi.org/10.1007/s12035-026-06202-2.

## Introduction

Spontaneous subarachnoid hemorrhage (sSAH), most commonly caused by rupture of an intracranial aneurysm, is a severe neurological condition associated with high mortality and long-term disability [1]. The initial hemorrhagic insult triggers a complex cascade of pathophysiological processes, including early brain injury, inflammation, cerebral vasospasm, delayed cerebral ischemia (DCI), brain edema, and occasionally rebleeding, ultimately leading to neurological deterioration and systemic multi-organ dysfunction. DCI, occurring typically between days 4 and 14 after hemorrhage, affects approximately 20–30% of patients and remains a major contributor to morbidity and poor outcome [1].

Despite advances in surgical, endovascular, and intensive care management, early identification of patients at higher risk of poor outcome or complications remains challenging. Current prognostic assessment relies mainly on clinical grading scales and neuroimaging findings, which may incompletely capture the underlying extent of biochemical and cellular brain injury, particularly in the early phase after hemorrhage [2–6].

In recent years, several circulating biomarkers have been investigated as potential tools for outcome prediction in sSAH [2, 4, 6]. Among them, S100-beta protein (S100B) has emerged as a promising indicator of neuronal and glial injury, with established prognostic value in traumatic brain injury [7, 8]. S100B is a calcium-binding protein predominantly expressed in astrocytes and released into the bloodstream following blood–brain barrier disruption and cerebral injury, allowing early peripheral detection. While elevated S100B levels have been reported in various forms of acute brain injury, including ischemic and hemorrhagic stroke, evidence in sSAH remains limited and heterogeneous, and its role in predicting disease progression, complications, and outcomes has not been clearly defined [7, 9–11].

The study aims to evaluate whether early serum S100B levels reflect the severity of early brain injury and predict functional outcomes, mortality, delayed cerebral ischemia (DCI), and systemic complications, primarily as downstream consequences of the initial hemorrhagic insult rather than as markers of delayed ischemic mechanisms themselves.

## Materials and Methods

### Study Design

This prospective observational study included all adult patients (> 18 years) admitted to the Emergency Department of Fondazione Policlinico Universitario A. Gemelli IRCCS (Rome) between January 2022 and August 2024 with a diagnosis of spontaneous subarachnoid hemorrhage (sSAH) confirmed by neuroimaging. Both aneurysmal sSAH and hemorrhage related to other vascular malformations were included [12, 13] while patients with traumatic subarachnoid hemorrhage or other intracranial pathologies were excluded. S100B reflects blood-induced brain injury and neuroinflammatory responses triggered by subarachnoid blood exposure, which may occur across a broad spectrum of clinical presentations regardless of the underlying vascular source of subarachnoid hemorrhage [12–15]. Otherwise, post-traumatic SAH represents a distinct biological entity with a different neurological mechanism of brain damage. The study aimed to evaluate the association between serum S100B levels, their temporal trends, and clinical outcomes and complications in sSAH. Blood samples were collected at predefined time points: 24 h (T0), 72 h (T1), and 7 days (T2) upon admission. Because the exact time of ictus could not be reliably established in all patients, admission time was used as the reference point for sample collection. Analyses involving T1 were performed using a 72-h landmark approach and included only patients alive at the scheduled sampling time. Patients who died before 72 h were excluded from T1-based models, and T1 values were not imputed because the biomarker was structurally unobservable after death. All DCI events occurred between hospital days 4 and 14 and therefore after T1 sampling. Consequently, the T1–DCI model evaluated the association between S100B measured at 72 h and subsequent DCI.

Demographic, clinical, and radiological data were prospectively collected from hospital records.

### Study Endpoints

#### Primary Outcomes

- Association between S100B serum levels at different intervals and functional outcome measured by mRS at 14 days and 3 months
- Association between S100B serum levels at different intervals and mortality
- Association between S100B serum levels at different intervals and delayed cerebral ischemia (DCI)

#### Secondary Outcomes

- Association between S100B levels and systemic complications (cardiac, renal, infectious, thromboembolic, and pulmonary events)

### Ethical Approval

The study was approved by the local Ethics Committee (ID FPG/6185) and registered at ClinicalTrials.gov (NCT06218654, 2024-01−15). Written informed consent was obtained from all participants or their legal representatives.

### Clinical and Radiological Data Collection

Baseline data included age, sex, Hunt and Hess scale, modified Fisher scale, blood volume on CT, vascular lesion type, cardiovascular risk factors, and mRS at admission. The use of external ventricular drainage, treatment modality (endovascular or surgical), and nimodipine administration were recorded [3, 16].

Length of hospital stay and mRS scores were assessed during hospitalization and at 14 days and 3-month follow-up.

All patients underwent CT imaging at diagnosis. Volumetric analyses of subarachnoid and intraparenchymal hemorrhage were independently performed by two blinded neuroradiologists. Intracranial complications classified as DCI, late hydrocephalus, rebleeding or cerebral edema requiring decompressive craniectomy, and systemic complications were recorded. Late hydrocephalus was defined as hydrocephalus developing after the acute phase of sSAH and requiring permanent cerebrospinal fluid diversion [1, 2, 4, 6, 17, 18]. Rebleeding was defined as a new post-treatment hemorrhagic event arising from the index vascular lesion, confirmed by radiological evidence of increased hemorrhage volume on follow-up imaging compared with baseline studies, with or without associated neurological deterioration [19].

DCI was defined as new neurological deterioration lasting ≥ 1 h, occurring between hospital days 4 and 14, after the standardized T1 measurement at 72 h, confirmed by angiography or CT angiography, and not attributable to other causes [20].

### Clinical Outcome and Intracranial Complications

Clinical outcomes were measured using the modified Rankin Scale (mRS) at 14 days and 3 months of follow-up post-sSAH. Median values of serum sampling were evaluated to predict outcomes. Other median trends were evaluated for intracranial and systemic complications.

### Selection and Measurement of S100B Biomarker

Serum S100B levels were measured using an electrochemiluminescence immunoassay (Elecsys S100B, Roche Diagnostics) with a measuring range of 0.02–30.0 µg/L and a cutoff of 0.10 µg/L [5, 21, 22]. Serum samples were assessed at 24 h (T0), 72 h (T1) and at 7 days (T2).

### Statistical Analysis

Continuous variables were reported as mean ± SD or median with interquartile range, as appropriate. Categorical variables were analyzed using Fisher’s exact test. Group comparisons were performed using Student’s *t*-test or Mann–Whitney *U* test. Logistic regression models were used to assess associations between S100B levels and outcomes, with exclusion of models affected by overfitting. Rebleeding and cerebral edema requiring decompressive craniectomy were analyzed descriptively only (frequencies and median S100B levels) and were not included as covariates or outcomes in the univariate or multivariable regression models, owing to the limited number of events (*n* = 12). T1 analyses were conducted using a 72-h landmark approach and included the 101 patients alive at 72 h with an available T1 measurement. Accordingly, the T1 models for 14-day and 3-month mRS were conditional on survival to the 72-h landmark, whereas the T1 mortality model evaluated deaths occurring after 72 h.

A random forest model was trained using S100B levels at T0, T1, and T2 to predict functional outcomes at 14 days and 3 months. Model performance was assessed using classification accuracy and area under the receiver operating characteristic curve, and feature importance was evaluated using Gini impurity reduction. Stratified cross-validation and sensitivity analyses excluding extreme values were performed. Statistical significance was set at *p* < 0.05.

## Results

### Patients’ Baseline Characteristics

One hundred and eight patients were enrolled, with an average age of 59 years. Females represented 61% of the cohort. The baseline characteristics and clinical course of the cohort have been presented in Table 1.

**Table 1:** Overall patients’ baseline characteristics and clinical outcomes

Variables	Values
No of patients	108
Age (mean ± SD/median [IQR])	59 ± 14.55 y/60.4 (51.2–69.3)
Sex (M:F, *n* [%])	42:66 (39%:61%)
Smoke	
Yes	32 (30%)
No	76 (70%)
Hypertension	
Yes	78 (72%)
No	30 (28%)
Hunt&Hess score	
1–3	69 (64%)
4–5	39 (36%)
Modified Fisher score	
1–2	13 (12%)
3–4	95 (88%)
SAH blood CT volumetry (median, IQR)	
Total blood (ICH + SAH)	19 (11–30) cm^3^
SAH blood	14.5 (7–20) cm^3^
Type of vascular malformation	
Aneurysmal	88 (81%)
Other spontaneous SAH	20 (19%)
mRS before resuscitation	
0–2	36 (33%)
3–5	72 (67%)
Treatment	
EVD placement	55 (51%)
Endovascular	75 (70%)
Microsurgical	33 (30%)
Overall complications	
Yes	68 (63%)
No	40 (37%)
Clinical vasospasm (as DCI)	
Yes	27 (25%)
No	81 (75%)
Other complications	
Late hydrocephalus	18 (17%)
Rebleeding/brain edema	12 (11%)
Systemic	34 (31%)
Length of stay (mean ± SD)	27.9 ± 46.7 d
mRS at 14 days	
0–2	54 (50%)
3–6	54 (50%)
mRS at 3-month follow-up	
0–2	65 (60.2%)
3–5	21 (19.4%)
6	22 (20.4%)
3–6	43 (39.8%)

*cm*^*3*^ cubic centimeters, *CT* computed tomography, *DCI* delayed cerebral ischemia, *EVD* external ventricular drainage, *F* female, *ICH* intracranial hematoma, *M* male, *mRS* modified Rankin Scale, *No* number, *SAH* subarachnoid hemorrhage, *SD* standard deviation, *y* years old

Sixty-four percent had a Hunt&Hess (HH) score between 1 and 3, and 88% had a modified Fisher score between 3 and 4. The median total blood volume on CT was 19 cm^3^, with 14.5 cm^3^ for subarachnoid hemorrhage. Aneurysmal SAH was found in 81%, while other non-aneurysmal malformations were 19%. At presentation, 33% of patients had mRS (before resuscitation) 0–2, while 67% had mRS 3–5. Regarding sSAH management, 70% underwent endovascular treatment and 30% underwent microsurgical treatment. Complications occurred in 63% of patients, with DCI in 25%. Late hydrocephalus affected 17%, and rebleeding or brain edema requiring DC affected 11%. Rebleeding or cerebral edema requiring decompressive craniectomy occurred in 12 patients (11%) and were analyzed descriptively only; no regression models were fitted for these events because of the limited number of cases. Systemic complications occurred in 31% of cases. At 14 days, functional outcome was dichotomized according to mRS, with 50% of patients presenting a favorable outcome (mRS 0–2). Three-month functional outcome data were available for all 108 enrolled patients. At 3 months, 65 patients (60%) achieved a favorable outcome (mRS 0–2), whereas 43 patients (40%) had an unfavorable outcome (mRS 3–6), including 22 patients who died during follow-up (mRS = 6, Table 1).

### S100B Levels and Functional Outcomes and Complications

**Fig. 1:**
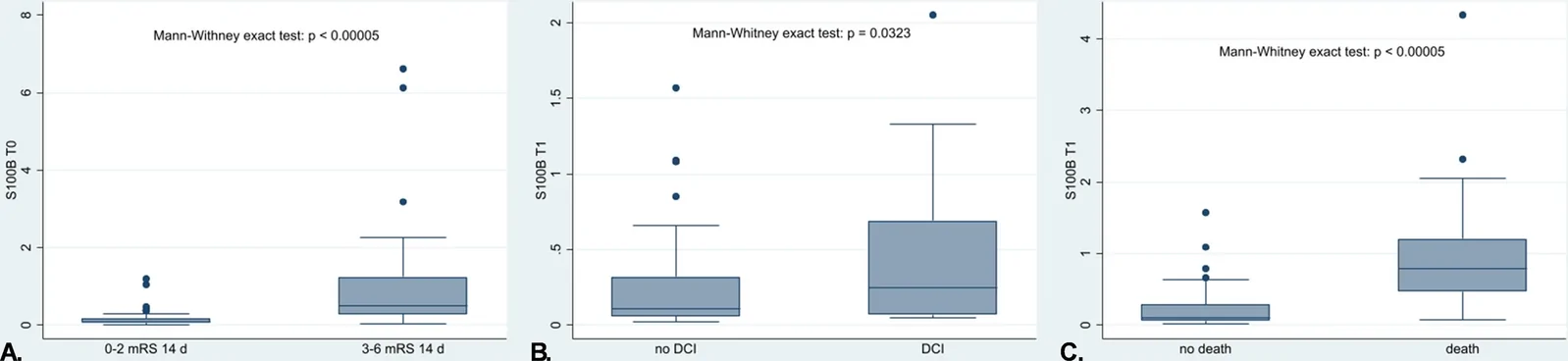
**A** Association between S100B levels at 24 h and 14-day dichotomized mRS. Boxplot with Mann–Whitney test. **B** Association between S100B levels at 72 h and DCI. Boxplot with Mann–Whitney test. **C** Association between S100B levels at 72 h and death. Boxplot with Mann–Whitney test

**Fig. 2:**
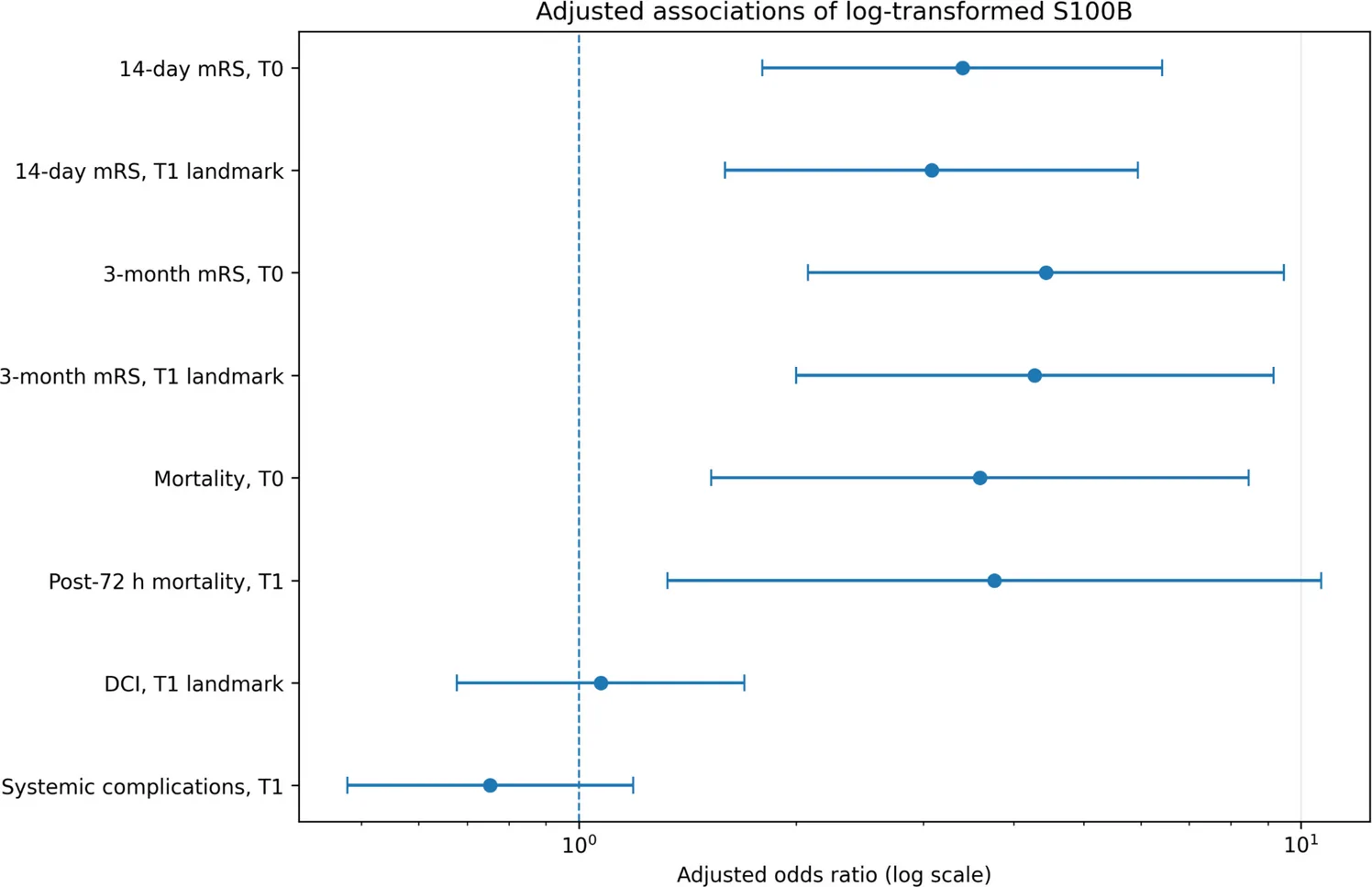
Adjusted associations between log-transformed serum S100B and clinical outcomes. Forest plot showing adjusted odds ratios and 95% CI from multivariable logistic regression models. All models were adjusted a priori for age, sex, and HH grade. T0 was measured at 24 h after admission and T1 at 72 h. T0 models were conducted as complete-case analyses and included 108 patients. T1 landmark models included 101 patients alive at 72 h with an available T1 measurement. The T1 models for 14-day and 3-month mRS were therefore conditional on survival to the 72-h landmark. The T1 mortality model evaluated deaths occurring after 72 h, and the T1 DCI model evaluated subsequent DCI occurring during hospital days 4–14. The vertical dashed line indicates an odds ratio of 1.0. DCI, delayed cerebral ischemia; mRS, modified Rankin Scale

Median S100B levels of 0.45 μg/L (IQR 0.26–0.79, Suppl. Table 1) at 24 h (T0) were associated with poor functional outcomes (mRS 3–6) at 3 months. Among the 108 patients with an available T0 measurement, higher log-transformed S100B levels at T0 were associated with unfavorable functional outcome at 14 days (OR 4.92, 95% CI 2.77–8.74, *p* < 0.001; Table 2, Fig. 1A) and at 3 months (OR 6.26, 95% CI 3.21–12.20, *p* < 0.001; Table 2). After adjustment for age, sex, and Hunt and Hess grade, S100B at T0 remained independently associated with unfavorable outcome at 14 days (adjusted OR 3.40, 95% CI 1.80–6.43, *p* < 0.001; Table 3, Fig. 2) and at 3 months (adjusted OR 4.43, 95% CI 2.08–9.47, *p* < 0.001; Table 3, Fig. 2). S100B was not independently associated with DCI in multivariable analysis (Table 3). T1 landmark analyses included 101 patients alive and evaluable at 72 h. The seven patients without a T1 measurement died before the scheduled 72-h sampling, and no patient alive at the landmark had a missing T1 measurement. Higher log-transformed S100B at T1 remained independently associated with unfavorable functional outcome at 14 days (adjusted OR 3.08, 95% CI 1.59–5.95, *p* < 0.001) and at 3 months (adjusted OR 4.28, 95% CI 2.00–9.17, *p* < 0.001). These analyses were conditional on survival to the 72-h landmark. All 27 DCI events occurred between hospital days 4 and 14, after T1 sampling. S100B at T1 was associated with subsequent DCI in univariable analysis (OR 1.60, 95% CI 1.06–2.41, *p *= 0.026, Fig. 1B), but the association did not persist after adjustment for age, sex, and Hunt and Hess grade (adjusted OR 1.07, 95% CI 0.68–1.70, *p* = 0.765; Tables 2 and 3, Fig. 2).

**Table 2:** Univariable logistic regression models for log-transformed S100B and clinical outcomes

14-day mRS, T0 (*n* = 108)	**Odds ratio**	***P* >|*z*|**	**[95% conf. interval]**	
S100B (T0)	4.92	** < 0.001**	2.77	8.74
14-day mRS, T1 landmark (*n* = 101)	**Odds ratio**	***P* >|*z*|**	**[95% conf. interval]**	
S100B (T1)	3.75	** < 0.001**	2.23	6.30
3-month mRS, T0 (*n* = 108)	**Odds ratio**	***P* >|*z*|**	**[95% conf. interval]**	
S100B (T0)	6.26	** < 0.001**	3.21	12.20
3-month mRS, T1 landmark (*n* = 101)	**Odds ratio**	***P* >|*z*|**	**[95% conf. interval]**	
S100B (T1)	5.22	** < 0.001**	2.78	9.79
Mortality, T0 (*n* = 108)	**Odds ratio**	***P* >|*z*|**	**[95% conf. interval]**	
S100B (T0)	5.28	** < 0.001**	2.55	10.94
Post-72 h mortality, T1 landmark (*n* = 101)	**Odds ratio**	***P* >|*z*|**	**[95% conf. interval]**	
S100B (T1)	6.71	** < 0.001**	2.67	16.85
DCI, T1 landmark (*n* = 101)	**Odds ratio**	***P* >|*z*|**	**[95% conf. interval]**	
S100B (T1)	1.60	**0.026**	1.06	2.41
Systemic complications, T1 landmark (*n* = 101)	**Odds ratio**	***P* >|*z*|**	**[95% conf. interval]**	
S100B (T1)	1.16	0.411	0.82	1.64

S100B values were log-transformed before analysis. Odds ratios refer to a one-unit increase in log-transformed S100B. T0 models were complete-case analyses and included 108 patients. T1 analyses used a 72-h landmark approach and included 101 patients alive and evaluable at 72 h. The T1 models for 14-day and 3-month mRS were conditional on survival to the 72-h landmark. The T1 mortality model included deaths occurring after 72 h, whereas the T1 DCI model evaluated subsequent DCI occurring between hospital days 4 and 14. All prespecified univariable associations corresponding to the multivariable models are reported irrespective of statistical significance

Bold entries indicate statistically significant results (*p* 0.05)

**Table 3:** Multivariable logistic regression models for log-transformed S100B and clinical outcomes

14-day mRS, T0 (*n* = 108)	**Odds ratio**	***P* >|*z*|**	**[95% conf. interval]**	
S100B (T0)	3.40	** < 0.001**	1.80	6.43
Sex (female)	0.98	0.973	0.27	3.51
Age (years)	1.04	**0.043**	1.00	1.09
HH grade	2.02	**0.006**	1.23	3.33
14-day mRS, T1 landmark (*n* = 101)	**Odds ratio**	***P* >|*z*|**	**[95% conf. interval]**	
S100B (T1)	3.08	** < 0.001**	1.59	5.95
Sex (female)	1.05	0.938	0.31	3.51
Age (years)	1.05	**0.025**	1.01	1.09
HH grade	2.19	**0.001**	1.37	3.51
3-month mRS, T0 (*n* = 108)	**Odds ratio**	***P* >|*z*|**	**[95% conf. interval]**	
S100B (T0)	4.43	** < 0.001**	2.08	9.47
Sex (female)	0.92	0.916	0.21	4.05
Age (years)	1.01	0.539	0.97	1.06
HH grade	2.68	**0.001**	1.47	4.90
3-month mRS, T1 landmark (*n* = 101)	**Odds ratio**	***P* >|*z*|**	**[95% conf. interval]**	
S100B (T1)	4.28	** < 0.001**	2.00	9.17
Sex (female)	0.84	0.808	0.20	3.47
Age (years)	1.02	0.388	0.98	1.07
HH grade	2.83	** < 0.001**	1.58	5.08
Mortality, T0 (*n* = 108)	**Odds ratio**	***P* >|*z*|**	**[95% conf. interval]**	
S100B (T0)	3.59	**0.003**	1.53	8.46
Sex (female)	1.80	0.542	0.27	11.98
Age (years)	1.03	0.384	0.96	1.10
HH grade	6.58	** < 0.001**	2.17	19.92
Post-72 h mortality, T1 landmark (*n* = 101)	**Odds ratio**	***P* >|*z*|**	**[95% conf. interval]**	
S100B (T1)	3.76	**0.013**	1.33	10.67
Sex (female)	1.32	0.818	0.12	14.25
Age (years)	1.08	0.093	0.99	1.18
HH grade	11.63	**0.004**	2.22	61.01
DCI, T1 landmark (*n* = 101)	**Odds ratio**	***P* >|*z*|**	**[95% conf. interval]**	
S100B (T1)	1.07	0.765	0.68	1.70
Sex (female)	1.26	0.659	0.46	3.45
Age (years)	0.99	0.657	0.96	1.03
HH grade	1.59	**0.024**	1.06	2.39
Systemic complications, T1 landmark (*n* = 101)	**Odds ratio**	***P* >|*z*|**	**[95% conf. interval]**	
S100B (T1)	0.75	0.223	0.48	1.19
Sex (female)	2.11	0.143	0.78	5.72
Age (years)	1.01	0.451	0.98	1.05
HH grade	1.73	**0.008**	1.15	2.58

S100B values were natural-log-transformed before analysis. Odds ratios refer to a one-unit increase in log-transformed S100B. All models were adjusted a priori for age, sex, and Hunt and Hess (HH) grade, T0 models were complete-case analyses (*n* = 108). T1 landmark models included the 101 patients alive at 72 h with an available T1 measurement. The T1 models for 14-day and 3-month mRS were conditional on survival to the 72-h landmark, and the T1 mortality model evaluated deaths occurring after 72 h. Deaths were classified as mRS 6 in the 3-month functional outcome models

Bold entries indicate statistically significant results (*p* 0.05)

Higher log-transformed S100B was associated with mortality at both time points. At T0, the univariable OR was 5.28 (95% CI 2.55–10.94, *p* < 0.001), and the adjusted OR was 3.59 (95% CI 1.53–8.46, *p* = 0.003). In the 72-h landmark analysis, S100B at T1 was associated with subsequent mortality in both univariable analysis (OR 6.71, 95% CI 2.67–16.85, *p* < 0.001, Fig. 1C) and multivariable analysis (adjusted OR 3.76, 95% CI 1.33–10.67, *p* = 0.013; Tables 2 and 3, Fig. 2). Nonparametric analysis using the Mann–Whitney *U* test did not demonstrate a statistically significant difference in S100B levels between patients with and without systemic complications, including cardiac events, infections, deep venous thrombosis, acute respiratory distress syndrome, and renal failure.

### Random Forest Model and Outcomes

**Fig. 3:**
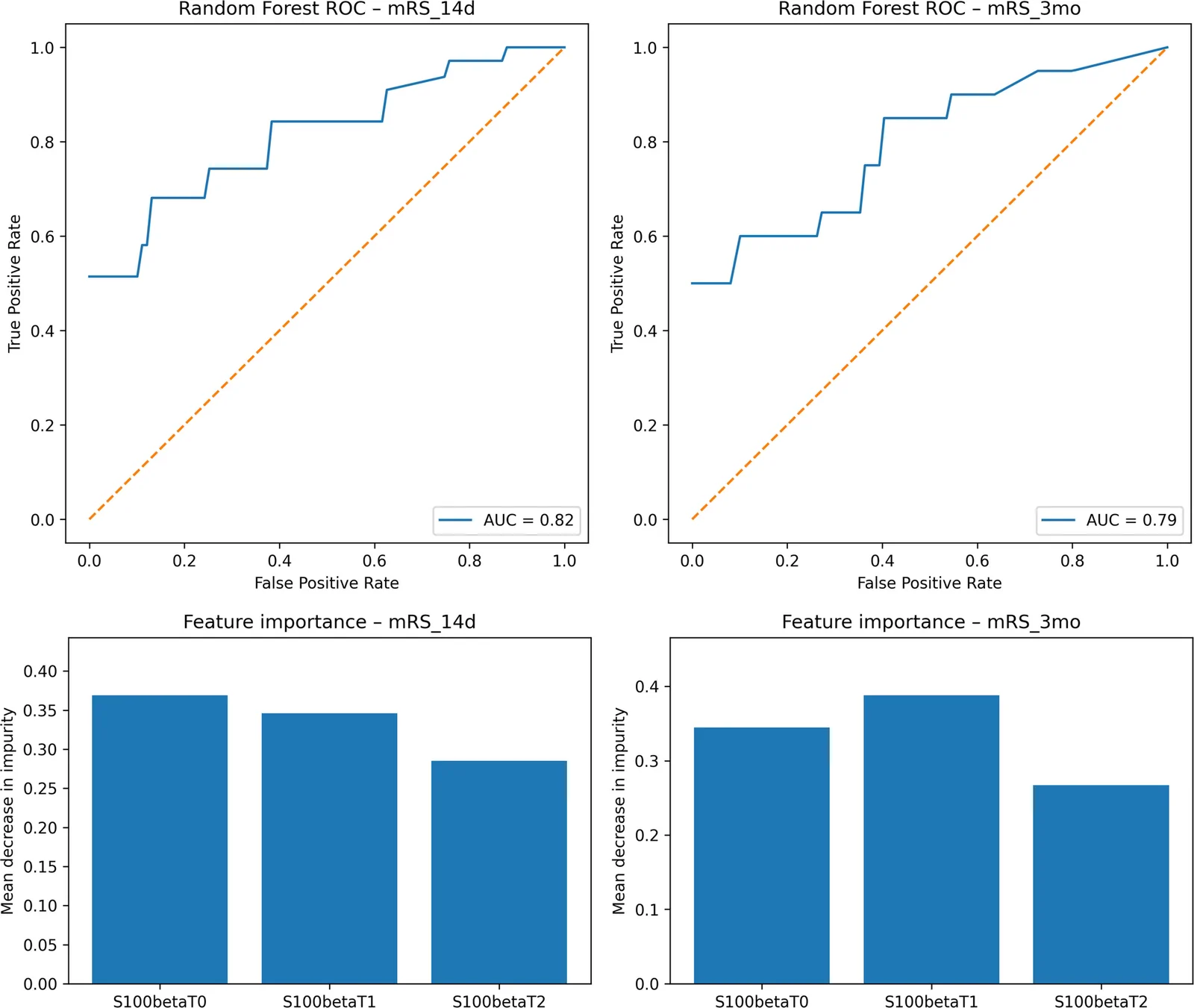
Random forest ROC curve and feature importance with S100B levels as predictor variables for mRS at 14 days (left side) and 3 months (right side). ROC curves generated from one representative train–test split. The AUC values displayed represent split-specific estimates and were not used as the primary measures of model performance

A random forest model including S100β levels at T0, T1, and T2 showed moderate discrimination for poor outcome at 14 days (cross-validated AUC ≈ 0.70–0.75) and at 3 months (AUC ≈ 0.68–0.73). Repeated stratified cross-validation demonstrated stable performance, with overlapping AUC distributions across folds (Fig. 3). Sensitivity analyses excluding extreme biomarker values yielded comparable results. The random forest model showed an accuracy of approximately 80% for 14-day outcome prediction and slightly lower performance for 3-month outcome (Fig. 3). As in the mRS prediction, S100B at T0 and T1 emerged as the most relevant predictors.

## Discussion

This study highlights the prognostic significance of serum S100B levels in patients with sSAH, demonstrating strong associations with functional outcomes and mortality. Our findings support the role of S100B as a biomarker reflecting the severity of early brain injury and its downstream clinical consequences, particularly when measured at early time points.

### Expanding Current Literature About S100B and DCI

The role of S100B as a biomarker in neurological disorders is well established, especially in traumatic brain injury and hypoxic–ischemic injury, where elevated serum levels reflect neuronal damage, blood–brain barrier disruption, and neuroinflammatory activation [1, 4]. Additionally, secondary increases in S100B have also been described after other acute cerebrovascular events, such as rupture of brain arteriovenous malformations, where higher levels have been associated with cerebral infarction, further supporting its role as a marker of ischemic brain injury across different neurological conditions [10]. Although the etiology of hemorrhage differed among patients, all included conditions shared the presence of spontaneous blood extravasation into the subarachnoid space, which is known to trigger similar inflammatory and neurotoxic pathways implicated in S100B release. However, the potential influence of sSAH etiology on biomarker kinetics warrants further investigation in larger homogeneous cohorts. In sSAH, previous studies have reported associations between S100B and DCI, although results have been heterogeneous and often limited by small sample sizes and insufficient adjustment for clinical severity (Suppl Table 2) [23]. In the present prospective observational study, we focused on the prognostic value of early S100B measurements obtained during the acute phase after sSAH and extended the analysis beyond DCI to encompass a broad spectrum of complications. Our results demonstrate that S100B is a strong predictor of functional outcome and mortality in patients with sSAH, particularly when measured at early time points (T0–T1). Although elevated S100B levels at T1 were associated with DCI in univariable analysis, this relationship did not persist after multivariable adjustment for clinical severity, suggesting that early S100B measurements may be more closely related to the severity of the initial brain injury than to delayed ischemic processes. Although Hunt and Hess grade was included in the multivariable models, other objective neuroradiological and clinical measures of early brain and vascular injury were unavailable. Therefore, residual confounding by unmeasured markers of cerebral edema and DCI cannot be excluded.

Taken together, these findings support the interpretation of S100B as a quantitative marker of global cerebral injury rather than a direct mediator or specific predictor of vasospasm or DCI. While established prognostic models based on clinical and radiological variables already provide robust risk stratification, the integration of biochemical biomarkers such as S100B may offer complementary information by capturing the biochemical burden of brain damage. Unlike clinical scales and imaging scores, which are partly categorical and observer-dependent, S100B provides a continuous and objective measure that may enhance prognostic discrimination among patients with similar clinical and radiological profiles. Accordingly, S100B should be considered not an alternative to established prognostic tools, but a complementary biomarker capable of refining prognostic assessment in sSAH.

### S100B as a Predictor of Functional Outcomes

The association between elevated S100B levels at T0 and unfavorable functional outcomes at both 14 days and 3 months supports its role as a marker of early brain injury severity following sSAH. S100B is released in response to astroglial injury, blood–brain barrier disruption, and neuronal damage, processes that occur during the acute phase after hemorrhage. Consequently, elevated S100B levels are likely to reflect the overall burden of brain injury rather than a specific pathological mechanism. This concept is analogous to the use of neurological biomarkers following cardiac arrest, where elevated biomarker levels reflect the extent of hypoxic–ischemic encephalopathy and are strongly associated with neurological outcome. Similarly, elevated S100B levels in SAH may identify patients who have sustained more severe early brain injury and are therefore at increased risk of disability and death (Table 3). Notably, S100B remained independently associated with unfavorable functional outcomes after adjustment for age, sex, and HH. These findings are also consistent with our previous study and further support the potential role of S100B as an adjunct tool for early risk stratification. Nevertheless, external validation in larger independent cohorts is required before routine clinical implementation [6]. A consistent finding across different studies is that an S100B level of approximately 0.20 µg/L predicts a poor functional outcome, as we also demonstrated in our previous works [6, 9].

### S100B and Mortality: A Biomarker for Severe Injury?

The association between S100B levels and mortality, particularly the persistence of the association in the T1 landmark model after multivariable adjustment (adjusted OR 3.76, 95% CI 1.33–10.67, *p* = 0.013), suggests that sustained S100B elevation may reflect severe neuronal injury, widespread ischemia, or persistent neuroinflammatory processes associated with poor survival. Given that mortality in sSAH is often influenced by a combination of early brain injury, secondary ischemic insults, and systemic complications, the extreme effect size observed at T1 suggests that S100B may be particularly relevant in identifying patients at imminent risk of neurological deterioration and death. Importantly, the high feature importance of S100B in the random forest predictive model further supports its potential role as a “prognostic biomarker.” However, this result should be interpreted considering that internal validation procedures were applied and the relatively limited sample size and the low number of events for some outcomes may have reduced model stability and increased the risk of overfitting. Therefore, these analyses should be considered exploratory and hypothesis-generating rather than directly applicable to clinical decision-making. Future studies involving larger, independent cohorts are required to validate the observed predictive patterns and determine their clinical relevance. The incorporation of S100B into predictive algorithms could enhance individualized risk stratification, potentially informing treatment escalation, intensive monitoring decisions, and even prognostic discussions with families.

### Strengths and Limitations of S100B

Unlike its strong association with neurological outcomes, S100B levels did not show a statistically significant relationship with systemic complications, including cardiac events, infections, deep venous thrombosis, acute respiratory distress syndrome (ARDS), or renal failure. While this may suggest that S100B remains predominantly a central nervous system-specific biomarker, although expressed at lower concentrations in adipose tissue, muscle, and melanocytes, it is also possible that systemic factors influencing its clearance and metabolism (e.g., renal and hepatic dysfunction) contribute to variability in its predictive value. Our results suggest that S100B may serve as a prognostic biomarker that, once validated in larger prospective and multicenter cohorts, could be integrated with established clinical and radiological parameters. Future identification and validation of clinically meaningful S100B thresholds may support more refined risk stratification and potentially inform clinical and therapeutic decision-making, particularly in patients with comparable clinical presentation and radiographic severity. The availability of a quantitative biomarker reflecting the extent of biochemical brain injury may be especially useful in the early phase after disease onset, when clinical assessment can be limited and radiological findings may not fully capture ongoing cellular damage. Importantly, the use of S100B should be considered complementary to standard clinical and radiological evaluation rather than a replacement. Further studies are warranted to confirm these findings, to define clinically meaningful cutoff values, and to assess whether the integration of S100B into existing prognostic models can improve their predictive performance and clinical utility before its implementation in routine practice. The absence of a significant association with systemic complications reinforces the specificity of S100B for intracranial injury and neurological outcome prediction.

## Limitations

This study has several limitations. The relatively limited sample size may reduce statistical power and restrict generalizability, although the cohort reflects a real-world sSAH population admitted to intensive care. The analysis was limited to biomarker measurements within the first 7 days, preventing evaluation of longer-term S100B dynamics. The timing of biomarker assessment was referenced to hospital admission rather than the exact time of hemorrhage, although patients were generally admitted shortly after symptom onset. Nevertheless, variability in the interval between ictus and admission may have introduced temporal bias in S100B measurements. Additionally, heterogeneity in clinical presentation, hemorrhage severity, and treatment strategies may have introduced residual confounding. The present study focused on the prognostic significance of early S100B measurements rather than longitudinal biomarker trajectories. Consequently, repeated-measures analyses were not performed. Furthermore, we recognize that the inclusion of the few non-aneurysmal sSAH cases may represent a potential confounding factor. Regarding the seven patients who died before the 72-h T1 sampling point, a descriptive comparison showed that they had higher initial severity (median Hunt and Hess grade 5 vs 3) and markedly higher S100B at T0 (median 1.58 vs 0.17 μg/L) than patients with an available T1 measurement. The exclusion of these patients limits the generalizability of T1-based findings to patients surviving to 72 h. Because the excluded patients had greater baseline severity, the observed associations may underestimate the prognostic information carried by persistent S100B elevation; however, the direction and magnitude of survivor-selection bias cannot be determined with certainty. Due to the unavailability of Subarachnoid Hemorrhage Early Brain Edema Score (SEBES) and the limited number of cases of rebleeding/brain edema, we could not assess whether the prognostic value of S100B was independent of objective markers of cerebral edema, which was defined using a clinically significant endpoint requiring decompressive craniectomy rather than a standardized radiological grading system. Although this approach identified patients with the most severe manifestations of edema, it may not have captured the full spectrum of early brain injury. Finally, the absence of long-term follow-up limited assessment of late functional recovery and delayed complications.

## Conclusion

This study supports the potential role of S100B as a prognostic biomarker for functional outcomes and mortality in patients with sSAH. The observed associations between S100B levels and unfavorable functional outcomes and mortality suggest that S100B may provide valuable information for early risk stratification. However, given the single-center design and the relatively limited sample size, these findings should be considered preliminary. Further validation in larger, independent cohorts is required before S100B can be incorporated into routine clinical practice or predictive models. Future research should also investigate the biological mechanisms underlying S100B elevation and assess its incremental value when combined with established clinical and radiological predictors.

## Supplementary Information

Below is the link to the electronic supplementary material.

ESM 1 (DOCX 16.1 KB)

ESM 2 (DOCX 30.0 KB)

## Data Availability

The datasets generated and/or analysed during the current study are available from the corresponding author upon reasonable request.

## Abbreviations

*AUC-ROC*: Area under the receiver operating characteristic curve
*CI*: Confidence interval
*CTA*: Computed tomography angiography
*DC*: Decompressive craniectomy
*DCI*: Delayed cerebral ischemia
*DICOM*: Digital imaging and communications in medicine
*ECLIA*: Electrochemiluminescence immunoassay
*EVD*: External ventricular drainage
*GCS*: Glasgow Coma Scale
*HH*: Hunt and Hess (Scale)
*IRB*: Institutional Review Board
*IQR*: Interquartile range
*IVH*: Intraventricular hemorrhage
*mRS*: Modified Rankin Scale
*OR*: Odds ratio
*sSAH*: Spontaneous subarachnoid hemorrhage
*T0*: 24 H after admission
*T1*: 72 H after admission
*T2*: 7 Days after admission
*TBI*: Traumatic brain injury
