# Supplementary material for: Serum S100B and Clinical Outcomes After Spontaneous Subarachnoid Hemorrhage: A Prospective Observational Study
Source: Mol Neurobiol. 2026 Sep 21;63(1):913. doi: 10.1007/s12035-026-06202-2 (PMC13593659; doi:10.1007/s12035-026-06202-2)
Supplement: Supplementary file 1 [file 12035_2026_6202_MOESM1_ESM.docx]

**Supplementary Table 1.** Median S100B levels across subgroups and outcomes.

|  | **S100B levels in 0-2mRS at 3Mo (median μg/L, IQR)** |  | | |
| --- | --- | --- | --- | --- |
|  | **T0** | 0.1 (0.06-0.18) | | |
|  | **T1** | 0.07 (0.05-0.2) | | |
|  | **T2** | 0.06 (0.03-0.10) | | |
|  | **S100B levels in 3-6mRS at 3Mo (median μg/L, IQR)** |  | | |
|  | **T0** | 0.45 (0.26-0.79) | | |
|  | **T1** | 0.33 (0.115-0.61) | | |
|  | **T2** | 0.18 (0.06-0.2) | | |
|  | **S100B levels in DCI (median μg/L, IQR)** |  | | |
|  | **T0** | 0.27 (0.11-0.73) | | |
|  | **T1** | 0.25 (0.07-0.69) | | |
|  | **T2** | 0.13 (0.04-0.28) | | |
|  | **S100B levels in Late Hydrocephalus (median μg/L, IQR)** |  | | |
|  | **T0** | 0.14 (0.08-0.76) | | |
|  | **T1** | 0.10 (0.06-0.55) | | |
|  | **T2** | 0.05 (0.03-0.20) | | |
|  | **S100B levels in Rebleeding/Edema (median μg/L, IQR)** |  | | |
|  | **T0** | 0.62 (0.31-1.56) | | |
|  | **T1** | 0.53 (0.32-1.09) | | |
|  | **T2** | 0.34 (0.11-0.54) | | |
|  | **S100B levels in Systemic complications (median μg/L, IQR)** |  | | |
|  | **T0** | 0.29 (0.16-0.54) | | |
|  | **T1** | 0.19 (0.06-0.43) | | |
|  | **T2** | 0.06 (0.04-0.2) | | |
|  | **S100B levels in Death (median μg/L, IQR)** |  | | |
| **T0** | | | 1.07 (0.54-1.58) |  |
| **T1** | | | 0.79 (0.46-1.26) |  |
| **T2** | | | 0.43 (0.26- 0.63) |  |

Values reported as median (IQR); non-parametric distribution
