# Supplementary material for: Serum S100B and Clinical Outcomes After Spontaneous Subarachnoid Hemorrhage: A Prospective Observational Study
Source: Mol Neurobiol. 2026 Sep 21;63(1):913. doi: 10.1007/s12035-026-06202-2 (PMC13593659; doi:10.1007/s12035-026-06202-2)
Supplement: Supplementary file 2 [file 12035_2026_6202_MOESM2_ESM.docx]

**Supplementary Table 2. Overview of Literature Examining the Relationship Between S100 and DCI**

| **Author, Year** | **Country** | **Number of patients** | **Type of study, Type of sample** | **CT Vasospasm/DCI** | **S100 and outcome** |
| --- | --- | --- | --- | --- | --- |
| Takayasu, 1985[24] | Japan | 16 | observational, CSF | DCI | Y |
| Persson, 1988[25] | Sweden | 45 | Prospective observational, serum | DCI | Y |
| Hårdemark, 1989[26] | Sweden | 32 | Prospective observational, CSF | DCI | Y |
| Schmid-Elsaesser, 2006[27] | Germany | 104 | Randomized Controlled Trial, serum | CTV and DCI | No report  (good and poor WFNS not compared each other) |
| Oertel, 2006[28] | Germany | 51 | Prospective observational, serum | CTV | Y |
| Weiss, 2006[7] | Germany | 74 | observational, serum | DCI | Y |
| Sanchez-Peña, 2008[29] | France | 109 | Prospective observational, serum | CTV/DCI | Y |
| Moritz, 2010[11] | Germany | 55 | Prospective observational, CSF | CTV/DCI | Y |
| Sanchez-Peña, 2012[23] | France | 278 | Comparative Study, serum | CTV/DCI | statin-treated and untreated patients |
| Zheng, 2017[30] | China | 105 | observational, serum | DCI | Y |
| Bründl, 2018[31] | Germany | 26 | Prospective observational, CSF | DCI | cognitive outcome in good-grade sSAH patients. |
| Garzelli, 2020[10] | France | 221 | Retrospective, serum | CTV | Y |

CSF: cerebrospinal fliud; CTV: CT vasospasm; DCI: delayed cerebral ischemia; sSAH: spontanenous subarachnoid hemorrhage; Y: yes

**References**

24. Takayasu M, Shibuya M, Kanamori M et al (1985) S-100 protein and calmodulin levels in cerebrospinal fluid after subarachnoid hemorrhage. J Neurosurg 63:417–420.https://doi.org/10.3171/jns.1985.63.3.0417

25. Persson L, Hårdemark H, Edner G et al (1988) S-100 protein in cerebrospinal fluid of patients with subarachnoid haemorrhage: a potential marker of brain damage. Acta Neurochir (Wien) 93:116– 122. https://doi.org/10.1007/BF01402892

26. Hårdemark HG, Almqvist O, Johansson T et al (1989) S-100 protein in cerebrospinal fluid after aneurysmal subarachnoid haemorrhage: relation to functional outcome, late CT and SPECT changes, and signs of higher cortical dysfunction. Acta Neurochir (Wien) 99:135–144.https://doi.org/10.1007/BF01402322

27 Schmid-Elsaesser R, Kunz M, Zausinger S et al (2006) Intravenous magnesium versus nimodipine in the treatment of patients with aneurysmal subarachnoid hemorrhage: a randomized study. Neurosurgery 58:1054–1065. https://doi.org/10.1227/01.NEU.0000215868.40441.D9 . (discussion1054-1065)

28. Oertel M, Schumacher U, McArthur DL et al (2006) S-100B and NSE: markers of initial impact of subarachnoid haemorrhage and their relation to vasospasm and outcome. J Clin Neurosci 13:834–840.https://doi.org/10.1016/j.jocn.2005.11.030

29. Zheng Y-K, Dong X-Q, Du Q et al (2017) Comparison of plasma copeptin and multiple biomarkers for assessing prognosis of patients with aneurysmal subarachnoid hemorrhage. Clin Chim Acta 475:64–69. https://doi.org/10.1016/j.cca.2017.10.009

30. Bründl E, Proescholdt M, Schödel P et al (2018) Excessive release of endogenous neuropeptide Y intocerebrospinal fluid after treatment of spontaneous subarachnoid haemorrhage and its possible impacton self-reported neuropsychological performance - results of a prospective clinical pilot study on good-grade patients. Neurol Res 40:1001–1013. https://doi.org/10.1080/01616412.2018.1508547
